# Assessing Residual Moisture After Sterilization as an Overlooked Source of Intraoperative Bacterial Contamination in Knee Intra-articular Reconstructions: Evaluating the Reliability of Routine Procedures and Tests

**DOI:** 10.1177/23259671241299409

**Published:** 2025-01-06

**Authors:** Konrad Malinowski, Michalina Bawor, Dong Woon Kim, Przemysław A. Pękala, Paweł Skowronek, Michael T. Hirschmann, Prof. Marcin Domżalski, Robert F. LaPrade, Marcin Mostowy

**Affiliations:** † Department of Anatomy, Jagiellonian University Medical College, Kraków, Poland; ‡ Artromedical Orthopedic Clinic, Bełchatów, Poland; § Medical University of Lodz, Lodz, Poland; ‖ Faculty of Medicine and Health Sciences, Andrzej Frycz Modrzewski Kraków University, Kraków, Poland; ¶ Department of Orthopaedic and Trauma Surgery S. Żeromski Hospital, Kraków, Poland; # Department of Orthopaedic Surgery and Traumatology, Kantonsspital Baselland (Bruderholz, Liestal, Laufen), CH-4101 Bruderholz, Switzerland University of Basel, CH-4051 Basel, Switzerland; ** Orthopedic and Trauma Department, Veteran’s Memorial Teaching Hospital in Lodz, Medical University of Lodz, Lodz, Poland; †† Twin Cities Orthopedics, Edina, Minnesota, USA; Investigation performed at the Artromedical Orthopedic Clinic, Bełchatów, Poland

**Keywords:** autoclave, epidemiology, infection, knee, knee ligaments, residual moisture, sterilization process

## Abstract

**Background:**

Contamination of sterilized surgical instruments is not a typically suspected source of increased infection rate, especially if no abnormalities in the sterilization process are detected.

**Purpose/Hypothesis:**

The purpose of this study was to report increased infection rates after knee ligament reconstructions due to undetectable sterilization process errors leading to residual moisture, not limited to a specific surgical tool. It was hypothesized that (1) residual moisture on surgical tools due to autoclave overloading would not be detected by autoclave self-diagnostics, chemical and biological tests, or organoleptic assessment and (2) this kind of contamination may elevate infection rates, especially in knee intra-articular reconstruction procedures.

**Study Design:**

Case series; Level of evidence, 4.

**Methods:**

A retrospective analysis of increased postoperative knee infection rate between January 2013 and January 2015 was performed. The inclusion criteria were all articular procedures. The exclusion criteria were joint arthroplasties, fractures, and open joint wounds. Criteria defining postoperative joint infections were as follows: (1) pain and effusion relapse and loss of achieved range of motion; (2) opaque/cloudy fluid on arthrocentesis; (3) fever >37.5°C lasting ≥3 days; and (4) ≥2-fold elevation in C-reactive protein levels, with symptoms onset within 21 days postoperatively. A negative culture result did not exclude a postoperative joint infection diagnosis and treatment. The data were summarized, and the infection rates of given subgroups were compared with a 2-tailed Fisher exact test. A risk ratio (RR) with 95% CIs was calculated.

**Results:**

Out of 533 orthopaedic procedures screened for inclusion, 4 joint arthroplasties, 7 fractures, and 2 open joint wounds were excluded. The remaining 520 articular procedures were included in the study—118 knee cruciate ligament reconstructions, 130 knee nonreconstruction arthroscopies, and 272 knee extra-articular/other joints arthroscopic and sports procedures. A total of 21.2% of knee intra-articular ligament reconstructions were complicated by postoperative joint infections, compared with 1.5% of knee nonreconstruction arthroscopies (RR, 13.8 [95% CI, 3.3-56.9]; *P* < .001) and 0.4% of knee extra-articular/other joints arthroscopic and sports procedures (RR, 57.6 [95% CI, 7.9-420.4]; *P* < .001). The source of the increased infection rate was identified as residual moisture on surgical tools due to autoclave overloading. This residual moisture was not detected by autoclave self-diagnostics, chemical and biological tests, or organoleptic assessment. After reducing the insert of surgical tools in the autoclave, the infection rate in the next 2 years after knee reconstructive procedures returned to <1% (*P* < .001).

**Conclusions:**

Our study demonstrated that residual moisture after the sterilization process may be an underestimated source of postoperative joint infections, undetectable in routine procedures and tests. Overcrowding of surgical equipment in the autoclave may be a root cause of this residual moisture identified. This kind of contamination may elevate the infection rate, especially in knee intra-articular reconstruction procedures.

Postoperative knee infections have been reported to occur in 0.19% to 0.28% of routine knee arthroscopy procedures^5,7^ and in 0.14% to 2.6% of anterior cruciate ligament reconstructions (ACLRs).^2,13,21^ However, recent studies have shown that bacterial deoxyribonucleic acid as a marker of subclinical infection may be present in as much as 85% of revision ACLRs even though the cultures were only positive in 3% of cases.^11,12^ The importance of those subclinical joint infections is not well understood; however, it was hypothesized to be an underestimated source of ACLR failure.^11,12^ Bacterial subclinical contamination was shown to decrease the mechanical strength of the grafts,^34^ increase tunnel widening, and increase the risk of ACLR failure, as confirmed by the presence of bacterial deoxyribonucleic acid (a marker of subclinical infection, more sensitive than culture results) in 85% of revision ACLRs compared with about 20% of primary ACLRs.^11,12^ A possible explanation is the formation of biofilm^12,15,34,37^ on the intra-articular grafts, 5- to 10-fold larger volume than other human joints^9,16,20,23^ and potentially lower effectiveness of antibiotic prophylaxis due to poor penetration of antibiotics from the blood vessels to the joint cavity.^29^

Contamination of sterilized surgical instruments is not a typically suspected source of increased infection rates. In the literature, there are some reports of bacteria identified in the surgical material that was believed to be sterile^24,35,36^; however, those studies demonstrated that it was due to the specific properties of given surgical tools that resulted in manufacturing process errors^36^ or human errors.^24,35^

Therefore, this study aimed to report on increased infection rates after knee ligament reconstructions due to undetectable sterilization process errors leading to residual moisture, not limited to a specific surgical tool. We hypothesized that (1) residual moisture on surgical tools due to autoclave overloading would not be detected by autoclave self-diagnostics, chemical and biological tests, or organoleptic assessment and (2) this kind of contamination may elevate the infection rate, especially in knee intra-articular reconstruction procedures.

## Methods

This study was approved by the Regional Bioethics Committee (approval No: 7/2023 from March 8, 2023) and conducted in accordance with the Helsinki Declaration of Ethical Principles for Medical Research with its latest amendments. All patients’ data were anonymized.

### Study Design and Criteria

This was a retrospective analysis of an epidemiological investigation conducted because of increased postoperative knee infection rates between January 2013 and January 2015 in the practice of a high-volume sports medicine surgeon (K.M.). The prolonged time from the increased infection rate period to this report is due to local legal regulations on operative complications. All patients operated on between January 2013 and January 2015 were screened for inclusion in the study. The inclusion criteria were all articular surgical procedures, both intra- and extra-articular. The exclusion criteria included joint arthroplasties, fractures, and open joint wounds. These criteria were identified to ensure a more homogeneous study group and enable comparisons between 3 specific categories: (1) knee intra-articular ligament reconstructions; (2) knee nonreconstruction arthroscopies; and (3) extra-articular/other joint arthroscopies and sports-related procedures. As the study analyzed the practice of a high-volume sports medicine surgeon, only single cases of arthroplasties, fractures, and open joint wounds were treated in the study timeframe; therefore, the exclusion criteria did not diminish the number of patients in a significant manner. All patients underwent standard perioperative antibiotic prevention, which in that timeframe was cefazolin administered intravenously half an hour before skin incision, in agreement with national guidelines.

The following criteria were utilized for a diagnosis of a postoperative joint infection: (1) relapse of pain and joint effusion and loss of achieved postoperatively stable level of pain and range of motion; (2) opaque/cloudy fluid in arthrocentesis; (3) fever >37.5°C lasting ≥3 days; and (4) ≥2-fold elevation in C-reactive protein (CRP) levels with onset of symptoms occurring within 21 days postoperatively. All these criteria had to be present together to diagnose a postoperative joint infection. If the criteria were met, 2 cultures were sampled at least twice in each case. An oral antibiotic regimen (therapeutic dose of clindamycin every 8 hours) was started in each case after sampling cultures. CRP levels were reassessed after 2 days of treatment; however, there was either a plateau or an increase in the CRP level in each case. The next step of treatment was intravenous administration of antibiotics and arthroscopic debridement.^26^ A negative culture result did not exclude a postoperative joint infection diagnosis, the same as in the studies of Viola et al^36^ and Parada et al.^24^

### Statistical Analysis

The data were summarized and the infection rates of given subgroups were compared using the Statistica 13.3 software (StatSoft), with a 2-tailed Fisher exact test. A risk ratio (RR), with 95% CIs, was calculated.

## Results

Documents and charts of 533 consecutive orthopaedic procedures between January 2013 and January 2015 were assessed. Four joint arthroplasties, 7 fractures, and 2 open joint wounds were excluded. The remaining 520 articular procedures were included in the study. This number consisted of 118 knee intra-articular ligament reconstructions (anterior/posterior cruciate ligaments), 130 knee nonreconstruction arthroscopies, and 272 knee extra-articular/other joint arthroscopies and sports-related procedures.

Out of the 118 patients who had undergone knee intra-articular reconstructions with the use of grafts or implants, 25 had developed a postoperative knee infection (21.2%). This was a significantly higher percentage than in patients with knee intra-articular nonreconstruction surgeries (1.5%) (RR, 13.8 [95% CI, 3.3-56.9]; *P* < .001), as well as in patients with knee extra-articular/other joint arthroscopies and sports-related procedures (0.4%) (RR, 57.6 [95% CI, 7.9-420.4]; *P* < .001), as specified in Table 1.

**Table 1:** Distribution of Postoperative Joint Infections^*a*^

	Infection, n	No Infection, n	Infection Rate, %	Comparison of Infection Rate With Knee Intra-articular Ligament Reconstructions
Knee intra-articular ligament reconstructions, n = 118	25	93	21.2	N/A
Knee nonreconstruction arthroscopies, n = 130	2	128	1.5	*P* < .001 13.8 [3.3-56.9]
Knee extra-articular/other joints arthroscopic and sports procedures, n = 272	1	271	0.4	*P* < .001 57.6 [7.9-420.4]

a Data are presented as n, %, or RR [95% CI]. N/A, not applicable; RR, relative risk.

Notably, culture results were positive in 5 out of the 25 cases. In 4 cases, coagulase-negative *Staphylococcus* culture was detected, with 2 additional cases identified as *Staphylococcus epidermidis* and *Escherichia coli* detected in 1 case. However, all criteria for joint infection occurred in all 25 cases: (1) pain and effusion relapse and loss of achieved range of motion; (2) opaque/cloudy fluid on arthrocentesis; (3) fever >37.5°C lasting ≥3 days; and (4) ≥2-fold elevation in CRP levels with symptoms onset within 21 days postoperatively.

**Figure 1.**
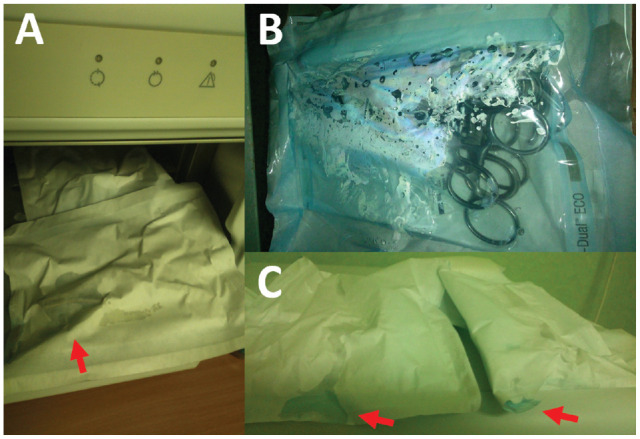
Evident traces of moisture in the supposedly sterile packs were observed in January 2015. (A) A pack of surgical tools. The red arrow shows a trace of moisture visible on the paper-like part of the pack. (B) A pack of surgical tools. Droplets of fluid are visible on the foil-like part of the pack. (C) Packs of surgical tools. Red arrows show traces of moisture visible on the paper-like part of the pack.

After a long epidemiological investigation, the source of the increased infection rate (21.2%) was identified as autoclave overloading, resulting in residual moisture on surgical tools. The novelty of this identification was that this residual moisture was not detected by autoclave self-diagnostics or chemical and biological tests. Furthermore, for most of the timeline described, sterilized surgical tool packs could not be reliably assessed by organoleptic methods. In January 2015, increased vigilance of the operating theater personnel resulted in the identification of the evident traces of moisture inside the supposedly sterile packs (Figure 1). The culture samples were taken from the tools and positive coagulase-negative *Staphylococcus* culture was cultivated. Notably, the autoclave self-diagnostics as well as chemical and biological tests were all negative, even though the evident traces of residual moisture were present.

Formal inspection of the autoclave and sterilization process was conducted and incidental overloading of the autoclave was identified. After reducing the inserts of surgical tools in the autoclave, the postoperative infection series ended with the infection rate in the next 2 years after knee reconstructive procedures returning to <1% (*P* < .001).

**Figure 2.**
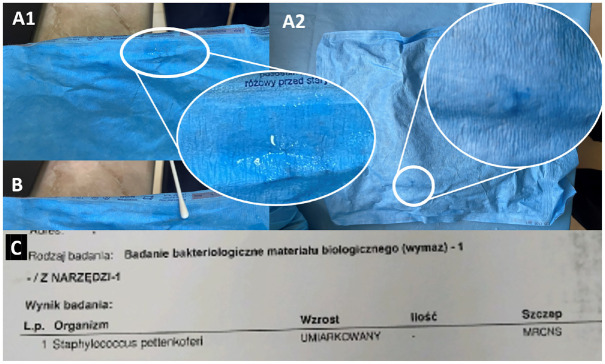
Assessment of moisture on the surgical tool packs with subsequent culture sampling. (A1 and 2) Packages of surgical tools with traces of moisture visible and magnified in the white circles. (B) Sampling the cultures from the packs. (C) Culture positive for *Staphylococcus* species growth.

In the following years after data collection, the residual moisture was observed once again in 2021 inside the supposedly sterile packs delivered from a different sterilization facility, proving that was not a single-facility problem (Figure 2A). The cultures were sampled from tools and packs (Figure 2B) and were positive for *Staphylococcus* species growth (Figure 2C). Notably, analogously (as in the described above case series), autoclave sterilization process self-diagnostics as well as biological and chemical tests were correct.

## Discussion

The most important result of this study was that residual moisture on surgical instruments caused by autoclave overloading was not detected by autoclave self-diagnostics, chemical and biological tests, or organoleptic assessment but instead elevated the complication rate, especially in knee intra-articular reconstruction procedures—21.2% versus 1.5% in knee intra-articular nonreconstruction surgeries (RR, 13.8 [95% CI, 3.3-56.9]; *P* < .001) versus 0.4% in knee extra-articular/other joints arthroscopic and sports procedures (RR, 57.6 [95% CI, 7.9-420.4]; *P* < .001).

After reducing the number of surgical tool inserts in the autoclave, the postoperative infection rate dropped to <1% (*P* < .001) over the following 2 years for knee reconstructive procedures. Out of the 118 patients who underwent knee intra-articular reconstructions with grafts or implants, 25 (21.2%) had previously developed a postoperative infection. This was a significantly higher percentage than in patients with knee intra-articular nonreconstruction surgeries (1.5%) (RR, 13.8 [95% CI, 3.3-56.9]; *P* < .001) as well as in patients with knee extra-articular/other joint arthroscopies and sports-related procedures (0.4%) (RR, 57.6 [95% CI, 7.9-420.4]; *P* < .001), as specified in Table 1.

Contamination of sterilized surgical instruments is not typically suspected as a source of increased postoperative infection rate, especially if no abnormalities in the sterilization process are detected. However, the abnormality that occurred in the described case series was a human-related error—namely, autoclave overloading leading to residual moisture on supposedly sterile surgical tools. It is important to note that the root cause was incidental autoclave overloading caused by a series of factors, including a “tight schedule” in a busy surgeon’s practice doing multiple intra-articular procedures in a day, the cost of running the procedure multiple times, or turnover time of 1 set of equipment, among other facts. This human-related error resulted in subtle residual moisture, which led to bacterial contamination and subsequently caused subclinical or clinical infections. Although numerous solutions to improve the reporting of human-related errors have been proposed,^30^ this type of error can be particularly hard to identify for psychological reasons. These include a strong belief in the reliability of the sterilization process, the counterintuitive nature of suspecting contamination when no errors are visibly detected, and the feelings of shame or reluctance among health care providers to report their own mistakes.^8,25,27,28^ There must be in-service education and policy regarding the best sterilization practice, and a team approach to optimize patient safety should be reviewed regularly. Nevertheless, in the setting of an increased infection rate, being aware of residual moisture on the surgical tools as one of the possible reasons for infections may save the clinician and patients from complications. To suspect the sterilization process error as a source of increased bacterial contamination rate requires “thinking outside the box,” as in a surgeon’s mind the sterilization process is “sacred.” It took us 2 years of tedious effort and numerous trials and errors of preventive measures to identify the human-related error of autoclave overloading as the root cause of the increased contamination rate.

A critic could argue that if residual moisture on surgical tools was a real problem, increased infection rates should occur more often in many different orthopaedic departments. The counterargument is that the postoperative joint infection rate was elevated only in knee intra-articular reconstructions that used grafts or implants, while the infection rate remained within normal ranges in other procedures. Therefore, the problem of residual moisture after the sterilization process may be especially important for the surgeons or departments where high-volume knee intra-articular reconstruction is performed. While the vancomycin presoaking was shown to greatly reduce the rate of postoperative knee joint infection after ACLR,^6,14,32^ its impact on the subclinical bacterial contamination is not well understood. Furthermore, due to increasing antibiotic resistance, vancomycin presoaking may be only a temporary solution.^1,14^ The authors theorize that the reason for the higher infection rate in the knee versus other human joints may be its 5- to 10-fold larger volume^9,16,20,23^ and potentially lower effectiveness of antibiotic prophylaxis due to poor penetration of the antibiotic from the blood vessels.^30^ The increased infection rate after intra-articular reconstructions using grafts or implants may be due to biofilm formation on the foreign tissue or material.^13,15,34,37^ The formation of biofilm was reported to increase the bacterial tolerance to antibiotics as much as 1000-fold compared with the planktonic form.^4,31^ In addition, an increasing number of *Staphylococcus epidermidis* perioperative infections have recently been reported in orthopaedics.^19,22^ This bacteria has a very high capacity to form biofilm, causing a large epidemiological infection risk in orthopaedic procedures that use implants or grafts.^10,17,19,38^ This is in agreement with the study by Clement et al,^7^ who analyzed 3 databases from the United States and reported increased infection rates after “high-complexity” procedures, such as ligamentous reconstruction, mosaicplasty, or meniscus repair in comparison with the less complex knee arthroscopies. However, this is the first study to our knowledge to report on increased exposure to bacterial contamination elevating infection rates only in knee reconstructive procedures.

In this case series, the factor that helped us determine the source of pathogens was the delivery of evidently wet surgical tool packs in January 2015. While visibly wet packs are rare, even slightly damp packs should be considered unsterile. This aligns with the guidelines of the American National Standards Institute, which provides a moisture assessment checklist and flowchart.^33^ The literature states the following reasons for residual moisture: poor steam quality; autoclave malfunction; inappropriate loading or overloading; too little time for the material to cool down to room temperature before taking it out of the autoclave; malfunction of wrapping materials; and inappropriate humidity or temperature in the sterile storage area.^3,18,33^ Some of the previously mentioned issues are human-related errors, often caused by working in a hurry, such as overloading the autoclave, not allowing adequate cooling time, or sterilizing the same set of surgical tools between consecutive similar surgeries.^33^ As a result of these events, scrub nurses now perform a mandatory collective visual assessment for any residual moisture in surgical tool packs, and there is a heightened awareness of early signs of increased infection rates, such as prolonged effusions that could indicate “low-grade infections.” We regularly educate the sterilization room staff, whose services we rely on, about the importance of avoiding autoclave overcrowding, and we are committed to maintaining strict standards in this area.

The third clinically relevant message is that negative knee culture results do not preclude the presence of a postoperative joint infection. Positive culture results were found in only 5 out of 25 (20%) patients with postoperative joint infection in our case series. This is comparable to the results reported by Viola et al,^36^ who found positive cultures in 2 out of their 11 cases (18.2%) of *Staphylococcus epidermidis* postoperative infections. Similarly, in the study by Parada et al,^24^ only 1 out of 5 cultures (20%) yielded positive results for *Staphylococcus epidermidis*, while in 2 other cases, coagulase-negative *Staphylococcus* (without the possibility for further identification) were detected.

There were some limitations of this study. First, it was a single-arm retrospective case series. Second, the genetic mapping of infectious agents was not performed. Third, we only have limited data as to a clinical decrease in the infection rate after the change of sterilization (<1%) (*P* < .001). Fourth, we did not assess the time of day or autoclave use during the day. Last, the available demographic data were scarce.

## Conclusion

Our study demonstrated that residual moisture after the sterilization process is an underestimated source of postoperative joint infections, undetectable in routine procedures and tests. Overcrowding of surgical equipment in the autoclave may be a root cause of this residual moisture identified. This kind of contamination may elevate the infection rate, especially in knee intra-articular reconstruction procedures.
